# Combination of cadherin-17 and SATB homeobox 2 serves as potential optimal makers for the differential diagnosis of pulmonary enteric adenocarcinoma and metastatic colorectal adenocarcinoma

**DOI:** 10.18632/oncotarget.18828

**Published:** 2017-06-28

**Authors:** Tingting Bian, Jinli Zhao, Jia Feng, Qing Zhang, Li Qian, Jian Liu, Daishan Jiang, Yifei Liu, Jianguo Zhang

**Affiliations:** ^1^ Department of Pathology, Affiliated Hospital of Nantong University, Nantong, Jiangsu, P.R. China; ^2^ Department of Radiology, Affiliated Hospital of Nantong University, Nantong, Jiangsu, P.R. China; ^3^ Department of Chemotherapy, Affiliated Hospital of Nantong University, Nantong, Jiangsu, P.R. China; ^4^ Department of Emergency Medicine, Affiliated Hospital of Nantong University, Nantong, Jiangsu, P.R. China

**Keywords:** pulmonary enteric adenocarcinoma, metastatic colorectal adenocarcinoma, cadherin-17, SATB homeobox 2, immunohistochemistry

## Abstract

**Background:**

Pulmonary enteric adenocarcinoma (PEAC), a rare type of non-small cell lung cancer, has similar histological and immunohistochemical morphology to colorectal adenocarcinoma. Cadherin-17 (CDH17) and SATB homeobox 2 (SATB2) immunoexpression have recently been demonstrated in colorectal adenocarcinoma. In this study, we evaluated the value of CDH17 and SATB2 in the diagnosis of pulmonary enteric adenocarcinoma and metastatic colorectal adenocarcinoma.

**Methods:**

A total of 13 PEAC cases and 27 metastatic colorectal adenocarcinoma cases were enrolled in our cohort study. We analyzed the expressions of CK7, CK20, CDX-2, villin, cadherin-17 (CDH17), and SATB homeobox 2 (SATB2) using immunohistochemistry. Staining intensity and percentage of positive-staining cells were recorded. Sensitivity and specificity values for immunostains, individually and in combination, were computed and compared.

**Results:**

Combining CDH17 and SATB2 resulted in high sensitivity (76.92%) and specificity (100%). In our study, the use of CK7+, napsin A+, TTF-1+, napsin A+TTF-1+ in combination with CDH17-/SATB2- had a higher area under the curve compared to the combination CDH17-/SATB2-. However, no significant differences were observed between the combination CDH17-/SATB2- and other combinations (P>0.05).

**Conclusions:**

In combination, CDH17 and SATB2 serve as potential optimal markers for the differential diagnosis of PEAC and metastatic colorectal adenocarcinoma.

## INTRODUCTION

Since Tsao et al. [1] first diagnosed primary pulmonary adenocarcinoma with enteric differentiation (PEAC), and increasing number of studies have reported on this condition. The International Association for the Study of Lung Cancer (IASLC), American Thoracic Society (ATS), and European Respiratory Society (ERS) proposed a new classification scheme for pulmonary adenocarcinoma in 2011, and defined PEAC as a rare type of invasive lung carcinoma [2]. PEAC was added to the 2015 World Health Organization classification of lung tumors, in which the challenges in differentiating between PEACs and pulmonary metastases from colorectal adenocarcinoma were highlighted [3, 4]. Histologically, PEAC is composed mainly of tall columnar cells arranged in an irregular acinar or cribriform pattern with extensive central necrosis, closely resembling the appearance of intestinal epithelial and colorectal carcinomas. Additionally, PEAC has been defined as a primary pulmonary adenocarcinoma with a predominant (>50%) colorectal-like component. Morphologically, PEAC has histological and immunohistochemical similarities to colorectal adenocarcinoma, while some PEACs are positive for immunohistochemical markers of enteric differentiation, such as CDX-2 and CK20/7. Tumors whose cells are negative for any intestinal protein markers, should be described as “lung adenocarcinoma with enteric morphology” rather than as enteric carcinoma of the lung. Villin protein expression may also be detected [5]. Because of the differences in treatment and prognosis between PEAC and metastatic colorectal adenocarcinoma, distinguishing these two diseases is necessary.

Cadherin-17 (CDH17), a structurally unique member of the cadherin super family, is a cell adhesion molecule, and is transcriptionally regulated by CDX-2. CDH17 plays an important role in cell adhesion, cell recognition, and maintaining normal development and morphology of tissues and organs. CDH17 is expressed in normal intestine, colon, pancreatic ductal epithelium, and in gastrointestinal adenocarcinoma [6, 7]. A recent study proved that CDH17 was more sensitive than CDX-2 in diagnosing primary or metastatic colorectal adenocarcinoma [8].

SATB homeobox 2 (SATB2) is the most sensitive and specific marker of colorectal adenocarcinoma and tumors with osteoblastic differentiation [9–11]. SATB2 is a nuclear matrix-associated transcription factor, and has important functions in the regulation of gene transcription and chromatin reorganization. The sensitivity of SATB2 in colorectal adenocarcinoma reaches 80%–97% [9, 11–13]. Based on the specificity and sensitivity of CDH17 and SATB2 for colonic and rectal tumors and their low expression in primary pulmonary tumors, we chose to compare CDH17, SATB2, CK7, napsin A, thyroid transcription factor (TTF-1), CK20, CDX-2, and villin expression in PEAC and metastatic colorectal adenocarcinoma.

## RESULTS

**Figure 1:**
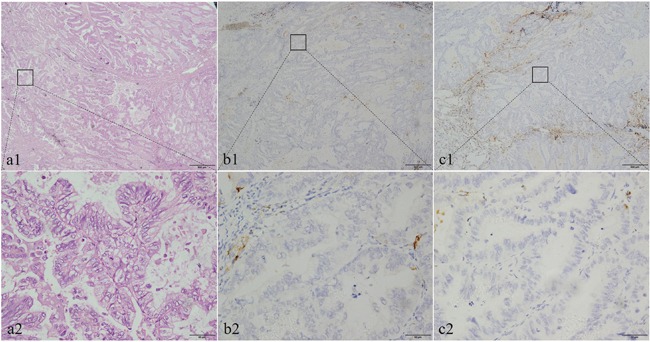
The histological morphology and the immunohistochemical expression of CDH17 and SATB2 in pulmonary enteric adenocarcinoma (PEAC) Histopathological morphology of pulmonary enteric adenocarcinoma shows in **(a1** and **a2)**. Negative IHC staining of CDH17 in PEAC samples **(b1, b2)**; negative IHC of SATB2 in PEAC samples **(c1, c2)**. Original magnifcation ×40 (scale bar 500μm) in **(a1, b1, c1)** and ×400 (scale bar 50μm) in **(a2, b2, c2)**.

**Figure 2:**
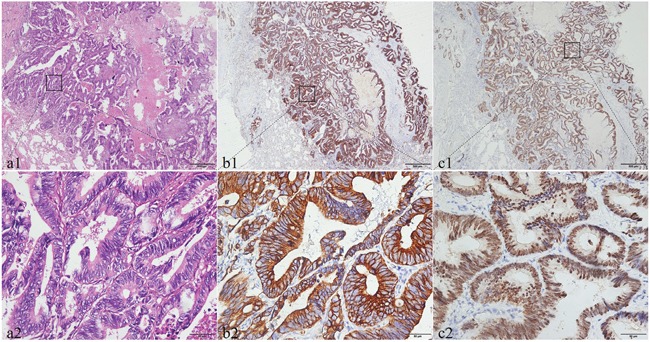
The histological morphology and the immunohistochemical expression of CDH17 and SATB2 in pulmonary metastatic colorectal adenocarcinoma Histopathological morphology of pulmonary metastatic colorectal adenocarcinoma shows in **(a1** and **a2)**. The immunohistochemical expression of CDH17 and SATB2 in pulmonary metastatic colorectal adenocarcinoma. The positive expression of CDH17 shows that the brown dye is distributed in the membrane **(b1, b2)**. The positive SATB2 expression with brown staining particles was distributed in cell nuclei **(c1, c2)**. Original magnifcation ×40 (scale bar 500μm) in **(a1, b1, c1)** and ×400 (scale bar 50μm) in **(a2, b2, c2)**.

Histologically, the neoplastic tissue was arranged in tubular or papillary patterns in PEAC. Some glandular structures contained a small amount of dust-like necrotic material. Cancerous tissues in columnar formations had abundant cytoplasm, and focal areas formed a brush border with increased nucleoplasmic ratio and mitotic figures (Figure 1). The histological morphology of pulmonary metastatic colorectal adenocarcinoma was very similar to that of PEAC (Figure 2).

Based on the pathological data of the 13 patients with PEAC (Table 1), we found the proportion of intestinal differentiation region to be 50%–85% and mitotic figures to occur at a rate of 2–9/10 HPF. Five cases exhibited neoplastic invasion of the pulmonary pleura. Eight cases were found to have necrosis of cancerous tissue, while six cases had airway infiltration. The maximum tumor diameter at surgery was 0.5–11 cm. PEAC exhibited histological heterogeneity and mixed histological subtypes of other lung adenocarcinomas, such as acinar and papillary structures. Comparisons of CT data including focus number, shape, boundary, lobulation, burr, bronchus sign, and pleural traction/ depression revealed no significant differences between the PEAC and metastatic colonic adenocarcinoma groups (P>0.05) (Table 2).

**Table 1:** Pathological features studies

No.	Size (cm)	Mitoses (10 HPF)	Visceral pleural invasion	Airway spread	Necrosis	Histologic patterns (%)				
						CR-like	Acinar	Papillary	Micro-papillary	Solid
1	1.5	3	-	+	-	50	0	0	50	0
2	2.0	3	+	-	-	80	20	0	0	0
3	0.8	4	-	-	-	70	20	10	0	0
4	2.0	7	+	-	-	50	40	10	0	0
5	0.6	2	-	-	+	85	10	5	0	0
6	3.5	4	+	-	+	60	20	20	0	0
7	5.0	3	+	+	+	80	20	0	0	0
8	3.0	2	+	+	+	70	15	15	0	0
9	11.0	9	-	+	+	60	30	10	0	0
10	0.5	4	-	-	+	60	20	20	0	0
11	4.5	2	-	+	+	50	20	20	10	0
12	3.0	7	-	+	-	50	50	0	0	0
13	2.5	8	-	-	+	80	20	0	0	0

HPF, high-power felids; CR-like, the colorectal carcinoma-like component.

**Table 2:** Comparison of CT features of pulmonary enteric adenocarcinoma and metastatic colorectal adenocarcinoma

CT features		PAEC	MCA	P value
Focus number	Single	11	23	1.000
	Multiple	2	4	
Shape	Round or oval	10	20	1.000
	Irregular	3	7	
Boundary	Clear	9	22	0.437
	Unclear	4	5	
Lobulated	Yes	8	19	0.722
	No	5	8	
Burr	Yes	10	22	1.000
	No	3	5	
Bronchus sign	Yes	5	6	0.451
	No	8	21	
Pleural retraction/ depression	Yes	8	13	0.511
	No	5	14	

PAEC, pulmonary enteric adenocarcinoma; MCA, metastatic colorectal adenocarcinoma.

The immunohistochemistry index of PEAC is shown in Table 3.

**Table 3:** Immunomarkers Profiles

No.	CK7	CK20	CDX-2	Villin	TTF-1	NapsinA	CDH17	SATB2
1	+++	-	-	+++	+++	+++	-	+
2	+++	-	-	+++	+++	+++	-	+
3	+++	-	-	-	-	-	+	-
4	+++	-	+++	+++	-	+++	-	-
5	-	+++	+++	-	+++	-	-	-
6	+++	++	+++	+++	-	-	-	-
7	+++	+++	-	+++	+++	-	-	-
8	-	+++	++	+++	-	+++	-	-
9	-	+++	+++	-	++	-	-	-
10	++	+++	+++	+++	-	-	-	-
11	+++	++	++	+++	+++	+++	-	-
12	+++	-	++	+++	-	+++	-	-
13	+++	+++	-	+++	+++	-	-	-

The immunohistochemical results for the 13 PEACs and, for comparison, the 27 patients with metastatic colorectal adenocarcinoma, are displayed in Table 4.

**Table 4:** Expression of immunohistochemical markers

IHC markers	PEAC (positive rate %)	MCA (positive rate %)	P value
CK7	10/13(76.9)	9/27(33.3)	0.038
CK20	8/13(61.5)	20/27(74.1)	0.476
CDX-2	8/13(61.5)	24/27(88.9)	0.086
Villin	10/13(76.9)	19/27(70.3)	1.000
NapsinA	6/13(46.2)	0	<0.001
TTF-1	7/13(53.8)	0	<0.001
CDH17	1/13(7.7)	20/27(74.1)	<0.001
SATB2	2/13(15.4)	20/27(74.1)	0.001

IHC markers, immunohistochemical markers; PAEC, pulmonary enteric adenocarcinoma; MCA, metastatic colorectal adenocarcinoma.

The rates of CDH17 and SATB2 positivity in PEAC were 7.7% (1/13) and 15.4% (2/13), respectively. Therefore, most cases of PEAC were not stained by immunohistochemical stains for CDH17 and SATB2 (Figure 1).

The rates of CDH17 and SATB2 positivity in metastatic colorectal adenocarcinoma were 74.1% (20/27) and 74.1% (20/27), respectively. Following immunohistochemical staining, the majority of cell membranes stained strongly positive for CDH17, as did most cell nuclei for SATB2 (Figure 2).

CK7-positive staining was identified in 76.9% (10/13) and 33.3% (9/27) of PEAC and metastatic colorectal adenocarcinoma samples, respectively. CK20-positive staining was identified in 61.5% (8/13) and 74.1% (20/27) of PEAC and metastatic colorectal adenocarcinoma samples, respectively. CDX-2-positive staining was found in 61.5% (8/13) and 88.9% (24/27) of PEAC and metastatic colorectal adenocarcinoma samples, respectively. Villin-positive staining was found in 76.9% (10/13) and 70.3% (19/27) of PEAC and metastatic colorectal adenocarcinoma tissues, respectively. Of the 13 cases with PEAC, six (46.2%) were positive for napsin A, and seven (53.8%) were positive for TTF-1. All 27 cases with colorectal adenocarcinoma were negative for napsin A and TTF-1.

In differentiating patients with PEAC from those with metastatic colorectal adenocarcinoma, the sensitivities (95% CI) of tissues staining CDH17-, SATB2-, napsin A+, TTF-1+, CK7+, and the combination of CDH17- and SATB2- were 92.31 (62.09–99.59), 84.62 (54.46–97.62), 92.31 (62.09∼99.60), 53.85 (26.12∼79.60), 76.92 (46.23∼94.71), and 76.92 (45.98–93.84), respectively. The corresponding specificities (95% CI) were 74.07 (53.70–88.82), 74.07 (53.70–88.82), 3.70 (0.19∼20.89), 96.30 (79.11∼99.81), 74.07 (53.70∼88.82), and 100 (84.50–100), respectively (Table 5).

**Table 5:** Sensitivity and specificity of immunostain combinations

Immunostain(s)	Sensitivity (95% CI), %	Specificity (95% CI), %
CDH17-	92.31(62.09∼99.59)	74.07(53.70∼88.82)
SATB2-	84.62(54.46∼97.62)	74.07(53.70∼88.82)
CK20+	69.23(38.88∼89.64)	18.52(7.03∼38.75)
CDX-2+	69.23(38.88∼89.64)	14.81(4.86∼34.61)
CK7+	76.92(46.23∼94.71)	74.07(53.70∼88.82)
Villin+	76.92(45.98∼93.84)	3.70(0.19∼20.89)
Napsin A+	92.31(62.09∼99.60)	3.70(0.19∼20.89)
TTF-1+	53.85(26.12∼79.60)	96.30(79.11∼99.81)
CDH17- and SATB2-	76.92(45.98∼93.84)	100(84.50∼100)
CK7+ and CDH17-	69.23(38.85∼89.64)	100(84.50∼100)
CK7+ and SATB2-	69.23(38.85∼89.64)	85.19(65.89∼95.14)
CK7+ CDH17- and SATB2-	58.85(26.12∼79.60)	100(84.50∼100)

CI, confidence interval.

The ROC curves for the immunohistochemistry markers were analyzed by logistic regression. The AUC (95% CI) for tissues staining CDH17-/SATB2-, CK7+/CDH17-/SATB2-, napsin A+/CDH17-/SATB2-, TTF-1+/CDH17-/SATB2-, napsin A+/TTF-1+/CDH17-/SATB2- were 0.950 (0.831–0.992), 0.994 (0.900–1.000), 0.990 (0.965∼1.000), 0.990 (0.965∼1.000), and 0.990(0.965∼1.000), respectively (P<0.01) (Table 6).

**Table 6:** Logistic regression: AUC demonstrating PEAC prediction

Immunostain(s)	AUC (95% CI)	SE	P value
CK7+	0.755(0.593∼0.877)	0.088	0.0098
CDH17-	0.832(0.680∼0.931)	0.068	0.0008
SATB2-	0.793(0.636∼0.905)	0.082	0.0029
NapsinA+	0.769(0.609∼0.887)	0.093	0.0064
TTF-1+	0.712(0.522∼0.902)	0.097	0.0013
Napsin A+ and TTF-1+	0.885(0.744∼1.000)	0.072	0.0001
Napsin A+ CDH17-and SATB2-	0.990(0.965∼1.000)	0.013	<0.0001
TTF-1+ CDH17- and SATB2-	0.990(0.965∼1.000)	0.013	<0.0001
Napsin A+ TTF-1+ CDH17- and SATB2-	0.990(0.965∼1.000)	0.013	<0.0001
CK7+ and CDH17-	0.940(0.817∼0.990)	0.035	<0.0001
CK7+ and SATB2-	0.859(0.712∼0.949)	0.055	<0.0001
CDH17- and SATB2-	0.950(0.831∼0.992)	0.044	<0.0001
CK7+ CDH17- and SATB2-	0.994(0.900∼1.000)	0.015	<0.0001

AUC, area under correlated receiver operating characteristic curve; CI, confidence interval.

A second logistic regression analysis demonstrated a significant difference between ROC curves for the combination CDH17-/SATB2- compared with other combinations. Specifically, the use of CK7+, napsin A+, TTF-1+, napsin A+ TTF-1+ in combination with CDH17- and SATB2- had a higher area under the curve compared with the combination of CDH17-/SATB2-. However, there was no significant difference observed between the combinations compared with the combination of CDH17-/SATB2- (P>0.05) (Table 7).

**Table 7:** Logistic regression: AUC demonstrating PEAC prediction compared with the combination of CDH17(−) and SATB2(−)

Immunostain(s)	Difference between areas	SE	95% CI	P value
CK7+	0.195	0.091	0.016 ∼ 0.374	0.033
CDH17-	0.118	0.058	0.004 ∼ 0.232	0.042
SATB2-	0.157	0.077	0.006 ∼ 0.307	0.041
Napsin A+	0.181	0.076	0.033∼0.329	0.017
TTF-1+	0.219	0.0778	0.067∼0.372	0.004
CK7+ and CDH17-	0.010	0.024	−0.036∼0.056	0.672
CK7+ and SATB2-	0.009	0.064	−0.033∼0.216	0.151
Napsin A+ and TTF-1+	0.066	0.057	−0.047∼0.178	0.254
Napsin A+ CDH17- and SATB2-	0.040	0.026	−0.012∼0.091	0.129
CK7+ CDH17- and SATB2-	0.044	0.043	−0.040 ∼ 0.128	0.301

SE, standard error; CI, confidence interval.

## DISCUSSION

Histologically, PEAC has features of intermediate differentiation, and sometimes forms a cribriform pattern, with tall columnar cells arranged in irregular acini or with extensive central necrosis. Tall columnar epithelial cells with eosinophilic cytoplasm, a brush border, and signet ring morphology may be also found in metastatic colorectal cancer. PEAC and metastatic colorectal adenocarcinoma, mainly occurring in middle-aged and elderly individuals, are significantly different in terms of treatment and prognosis. Hence, distinguishing the two diseases is of critical importance. The present study identified no significant gender predisposition. The right upper lung was affected in 53.8% of cases. A total of 76.9% patients had a history of smoking, which may be one potential risk factor for PEAC. Four patients died during follow-up, and the follow-up period was 6–9 months. Comparing the CT data of the 13 PEAC cases and 27 cases with metastatic colorectal adenocarcinoma, number of foci, shape, boundary, lobulation, burr, bronchus sign, and pleural traction/depression were not significantly different. Therefore, CT findings appear to be poorly specific for differentiating between PEAC and metastatic colorectal adenocarcinoma, and pathological examination is necessary for differential diagnosis of the two diseases. PEAC is a rare variant of adenocarcinoma, and its prognosis remains unclear.

Our study found that combining CDH17 and STAB2 in immunohistochemical staining could accurately distinguish PEAC and metastatic colorectal adenocarcinoma. The rate of CDH17-positivity in metastatic colorectal adenocarcinoma was 74.1% (20/27), but only 7.7% (1/13) in PEAC. Previous studies have proven that CDH17 is a highly sensitive marker of gastrointestinal adenocarcinoma and neuroendocrine tumors, and is rarely expressed in other tissues [6, 8]. Panarelli et al. found that the positive expression of CDH17 in colon cancer was 100% (161/161) [8]. Lin's study reported that the rate of CDH17-positivity was 98% (123/125) in colonic carcinomas [13]. Su et al. reported high expression rates (96%, 46/48) of CDH17 in colorectal adenocarcinoma, but that it was not expressed in pulmonary adenocarcinoma [6]. All of the results above are consistent with our study, in which we identified one case positive for CDH17 among the 13 cases with PEAC. We found that the positive expression of CDH17 in this case was weak, and concentrated at focal points, in comparison with the strong positive staining in metastatic colorectal adenocarcinoma. This weak positivity may be caused by tissue variation or differentiation of a particular subtype. However, CDH17 is expressed in some NSCLS, neuroendocrine tumors of bronchi, and endometrioid carcinoma [8]. Recent studies reported that CDH17 was a highly sensitive marker for bladder cancer and was positively expressed in 81% of metanephric adenomas [16, 17]. However, these tumors may be distinguished from gastrointestinal tumors based on morphology. Hence, CDH17 is highly sensitive for gastrointestinal tumors.

SATB2 is selectively expressed in the lower gastrointestinal tract mucosa and has been identified as a sensitive marker for colorectal cancer. Low expression in other tumors has proved the high specificity of SATB2 in colorectal cancer, and it serves as an important marker to distinguish primary colorectal cancer from metastatic tumors [9]. Previous studies have shown 80%–96.8% diffuse positive or strong positive SATB2 staining in colorectal cancers [7, 9, 11–13]. Magnusson reported that 85% of colorectal adenocarcinomas expressed SATB2, but that expression was rare in pancreatic cancer and gastric adenocarcinoma. In another study, the SATB2-positive rate in colorectal adenocarcinoma was 97%, but expression was rare in pancreatic cancer and upper gastrointestinal adenocarcinoma, and SATB2 expression in esophageal adenocarcinoma, gastric adenocarcinoma, and pancreatic adenocarcinoma was only 6.7%, 0%, and 4.2%, respectively [13]. Our study found that SATB2 expression rates in PEAC and metastatic colorectal adenocarcinoma were 15.4% (2/13) and 74.1% (20/27), respectively. Although highly sensitive, SATB2 is weakly positive in focal areas of some other tumors, such as pulmonary adenocarcinoma, pulmonary squamous carcinoma, bladder and ureteral tumors, and adenocarcinoma of the cervix, endometrium, and ovary [13]. We also found weak positive SATB2 staining in focal areas of PEAC. Recent studies have reported that decreased expression of SATB2 was related to poor prognosis and metastasis [18, 19]. One of the problems with our study is that the positive CDH17 and SATB2 rates are lower than those reported in the past. One possible reason for this finding is the small number of samples in the present study. A second reason may be changes in the microenvironment of the tumor. Therefore, the rates of CDH17 and SATB2 positivity in primary tumors may be higher than those of metastatic lesions.

Generally, CK20+, CK7-, and CDX-2+ statuses are considered to be markers of colorectal adenocarcinoma [20–24]. CK7 is expressed in many tumors, but has weak specificity [24]. Dragomir et al. [10] reported that the positive rate for CK7 in colorectal cancer tissue was 10%–27%. CK20 was expressed in tumors of some specific tissues, such as glandular epithelium of the gastrointestinal tract, urethral epithelium, and Merkel cells. Although CK20 had high sensitivity, it was not useful when used in isolation. The specificity of CK20 in metastatic colorectal adenocarcinoma was 65%–88% [8]. CK20 has often been combined with CK7 to diagnose different types of tumor. Our study showed that CK20 and CK7 were expressed in PEAC and metastatic colorectal adenocarcinoma, but the specificity of CK20, CK7, and villin were low. CDX-2 is intestine-specific, and immunohistochemical staining for this transcription factor has been proven able to determine whether colorectal cancer is primary or metastatic [21, 22]. Although CDX-2 is highly expressed in colorectal cancer tissues, it lacks specificity and may also be expressed in esophageal, gastric, and pancreatic cancer cells [22, 25]. A growing number of researchers have begun to focus on SATB2 for its improved sensitivity over CDX-2 in intestinal tumors [9, 12]. Recent studies have shown that CDH17 is more sensitive than CK20 and CDX-2 in the diagnosis of colorectal cancer [6, 7]. Brandler et al. also found that SATB2 had higher sensitivity, as compared with CDH17 [26]. A previous study found that CDH17 had higher sensitivity, but lower specificity than SATB2 [7]. The combination of CDH17 and SATB2 had high sensitivity in colorectal cancer with medullary features [13]. Our study found that CDH17 and SATB2 may have utility in the differential diagnosis of PEAC and metastatic colorectal adenocarcinoma, because they are highly expressed in metastatic colorectal adenocarcinoma but rarely expressed in pulmonary adenocarcinoma.

It is important to note that combinations of markers may be beneficial to clinical diagnosis. In the differential diagnosis of PEAC and metastatic colorectal adenocarcinoma, the sensitivities (95% CI) of CDH17-, SATB2-, and the combination CDH17-/SATB2- were 92.31 (62.09–99.59), 84.62 (54.46–97.62), and 76.92 (45.98–93.84), respectively. The corresponding specificities (95% CI) were 74.07 (53.70–88.82), 74.07 (53.70–88.82), and 100 (84.50–100), respectively. ROC curves were used to further verify this result. We compared the diagnostic value of CDH17-/SATB2- in PEAC and metastatic colorectal adenocarcinoma and we found the area under the ROC curve of the individual indices was smaller than the combined indices. In our study, the use of CK7+, napsin A+, TTF-1+, napsin A+ TTF-1+ in combination with CDH17- and SATB2- had a higher area under the curve compared with the combination CDH17-/SATB2-. However, there was no significant difference observed between the combinations compared with the combination of CDH17- and SATB2- (P>0.05). However, statistical analysis revealed the combination of CDH17- and SATB2- was not significantly more useful than other combinations. The reason for this may be the small sample size. As shown in Table 4, in PEAC, the rates of napsin A and TTF-1 positivity were only 46.2% (6/13) and 53.8% (7/13). This indicates that these two markers for PEAC may sometimes be negative. Both of these two markers were negative in all colorectal adenocarcinomas in our study. However, previous studies have shown that gastrointestinal tumors may also be positive for napsin A [27]. Thyroid transcription factor (TTF)-1 is expressed in the majority of pulmonary adenocarcinomas, but has only rarely been reported in adenocarcinomas originating at other sites, including colorectal adenocarcinoma [28, 29]. In our cases, the rates of CDX-2 positivity in PEAC and metastatic colorectal adenocarcinoma were 61.5% (8/13) and 88.9% (24/27), respectively. Previous studies have shown PEAC cells to be positive for at least one intestinal marker (such as CDX2, CK20, and MUC2) [30]. Other studies have also shown that 40% of PEAC were positive for CDX-2 [31]. As shown in Table 3, two PEAC cases were CDX-2+/TTF1-/napsin A-. Therefore, we hypothesized that the combination of CDH17- and SATB2- have potential as markers for the differentiation of PEAC and metastatic colorectal adenocarcinoma.

With advances in molecular technology, Wang et al. [30] detected nine cases of PEAC, and found that all of the tumors were EGFR-wild and KRAS-wild types. Laszlo et al. [31] demonstrated that mutations in the KRAS gene could distinguish PEAC from metastatic colorectal adenocarcinoma. Stojsic et al. [32] also reported that one of two cases of PEAC was KRAS-mutated. Alessia [4] found that PEACs exhibited a high frequency of KRAS mutations (60.9%), despite a low incidence of EGFR gene mutations (2.2%). Only a single case of PEAC had both EGFR and KRAS mutations. In addition, BRAF mutation was not found in any case. The lack of molecular analysis is a deficiency in our research. In subsequent studies, we will use genetic testing to discover the mechanisms underlying neoplastic mutation. We believe molecular analysis will become more important in future research. Gene expression profiling will become one method of choices to determine the origin of a tumor, especially in the case of small tissue samples.

Our study found that the combination CDH17-/SATB2- had high sensitivity (76.92%) and specificity (100%), and is a potential optimal marker for the differential diagnosis of PEAC and metastatic colorectal adenocarcinoma.

## MATERIALS AND METHODS

Surgically obtained lung cancer specimens, collected from the Affiliated Hospital of Nantong University between January 2009 and October 2016, were examined. This investigation was performed after obtaining patients’ informed consent and was approved by the local Human Research Ethics Committee in the Affiliated Hospital of Nantong University. Based on the new lung adenocarcinoma classification system established in 2011, the 2015 World Health Organization classification of lung tumors, and diagnosis by two experienced pathologists, 13 PEAC cases were enrolled in this cohort study. The 13 cases included six men and seven women (male:female, 1:1.17), with an age range of 47 to 80 years old, and an average age of 62.6 years. Ten patients had a history of smoking, while three did not. Tumors were located in the right upper lung, left upper lung, and left lower lung of seven, four, and two patients, respectively. The samples included three needle biopsies and ten lobectomy specimens, with the lobectomy specimens including four stage IA tumors, two stage IB, three stage IIA, and one stage IIB. All patients were followed-up to December 31st, 2016. Four patients died and nine survived, with survival times of 1–12 months. Clinical symptoms included a cough, expectoration, hemoptysis, and chest tightness without an apparent cause. A small number of patients experienced sustained chest and back pain. Clinical data of the 13 PEAC patients are shown in Table 8. In addition, 27 patients with metastatic colorectal adenocarcinoma were enrolled in the same cohort study. All tumors were staged according to the pathological tumor/node/metastasis (pTNM) classification (7th edition) of the Union for International Cancer Control [14].

**Table 8:** Clinical data

No.	Age/Sex	Location	Surgery	Smoking	p-Stage	Follow-up (Mo)
1	56/M	RUL	S	Yes	IA(T1a N0M0)	A(12)
2	53/F	RUL	S	No	IA(T1a N0M0)	A(12)
3	80/M	LUL	AB	Yes	NO	D(8)
4	69/F	LLL	S	Yes	IA(T1a N0M0)	A(6)
5	74/M	RUL	AB	Yes	NO	D(6)
6	56/M	RUL	S	Yes	IB(T2a N0M0)	A(7)
7	47/F	LUL	S	Yes	IIA(T2b N0M0)	A(8)
8	59/F	LLL	S	No	IB(T2a N0M0)	A(5)
9	61/M	RUL	S	Yes	IIB(T3N0M0)	D(8)
10	68/F	RUL	AB	No	NO	D(9)
11	60/F	LUL	S	Yes	IIA(T2a N0M0)	A(2)
12	77/M	RUL	S	Yes	IA(T1b N0M0)	A(2)
13	54/F	LUL	S	Yes	IIA(T2a N1M0)	A(1)

M, male; F, female; RUL, right upper lobe; LLL, left lower lobe; LUL, left upper lobe; AB, aspiration biopsy; S, segmentectomy; Mo, month; D, died; A, alive.

### Immunohistochemical staining

For H&E and immunohistochemical EnVision staining, lung tissues were embedded in paraffin and cut into 4 μm slices. The CK7 antibody (OV-TL12/30; 1:300) used in the study was produced by Zymed(San Diego, USA). CK20 (PW30; 1:100), TTF-1 (Sp T24; 1:200), and CDX-2 (AMT28, 1:50) antibodies were obtained from Novocastra(London, UK). Villin antibody (CWWB1; 1:100) and napsin A polyclonal antibody were obtained from Maixin (Fuzhou, China). CDH17 (HPA023616; 1:100) and SATB2 (HPA026871; 1:100) antibodies were obtained from Sigma (Shanghai, China). Blank controls were treated with PBS. The results were observed under a microscope.

### Staining results

In examining the prepared histological specimens, five randomly chosen high-power fields (100 cells/visual field) were visualized. The percentage of positively staining tumor cells was recorded. Cells were considered to be positive for CK7, CK20, or villin when the cytoplasm stained a brownish-yellow color. The cytoplasm of napsin A-positive cells contained brownish-yellow stained particles. Brownish-yellow nuclear particles were seen in cells staining positive for TTF-1, CDX-2, and SATB2. Cells were defined as CDH17-positive when the cell membrane stained a brownish-yellow color. Samples containing non-staining cells were classified as negative (−), < 25% positive-staining tumor cells as weak positive (+), 25%–75% as positive (++), and > 75% as strong positive (+++).

### Statistical analysis

The sensitivity and specificity of staining for CDH17, SATB2, CK7, CK20, CDX-2, and villin, with 95% exact binomial confidence intervals (95% CIs), were calculated. Our immunostain criterion for the diagnosis of metastatic colorectal adenocarcinoma were positive staining for CDH17 and SATB2, while for PEAC it was negative staining for CDH17 and SATB2. The differences between rates were tested by χ^2^ or Fisher's exact tests, if appropriate.

Logistic regression was used to model PEAC as a function of immunostaining. The corresponding receiver operating characteristic (ROC) curves were plotted for different combinations of immunostains, and the areas under these correlated ROC curves were compared using the nonparametric approach of DeLong et al [15]. All analysis was performed using SPSS statistical software (version 21.0) and MedCalc (version 9.2.1.0).
